# Developing a risk management framework to improve public health outcomes by enumerating and serotyping *Salmonella* in ground turkey

**DOI:** 10.1017/S0950268823002029

**Published:** 2024-01-08

**Authors:** Fernando Sampedro, Francisco Garcés-Vega, Ali J. Strickland, Craig W. Hedberg

**Affiliations:** 1 Environmental Health Sciences Division, School of Public Health, University of Minnesota, Minneapolis, MN, USA; 2 Independent Consultant, Cali, Colombia

**Keywords:** public health microbiology, prevention, risk assessment, *Salmonella*, turkey

## Abstract

*Salmonella enterica* continues to be a leading cause of foodborne morbidity worldwide. A quantitative risk assessment model was developed to evaluate the impact of pathogen enumeration and serotyping strategies on public health after consumption of undercooked contaminated ground turkey in the USA. The risk assessment model predicted more than 20,000 human illnesses annually that would result in ~700 annual reported cases. Removing ground turkey lots contaminated with *Salmonella* exceeding 10 MPN/g, 1 MPN/g, and 1 MPN/25 g would decrease the mean number of illnesses by 38.2, 73.1, and 95.0%, respectively. A three-class mixed sampling plan was tested to allow the detection of positive lots above threshold levels with 2–6 (*c* = 1) and 3–8 samples per lot (*c* = 2) using 25-g and 325-g sample sizes for a 95% probability of rejecting a contaminated lot. Removal of positive lots with the presence of highly virulent serotypes would decrease the number of illnesses by 44.2–87.0%. Based on these model prediction results, risk management strategies should incorporate pathogen enumeration and/or serotyping. This would have a direct impact on illness incidence linking public health outcomes with measurable food safety objectives, at the cost of diverting production lots.

## Introduction

Non-typhoidal *Salmonella* species are responsible for an estimated 1.2 million human illnesses, 23,000 hospitalizations, 450 deaths, and approximately $365 million in direct medical costs annually in the USA [1]. Rates of *Salmonella* cases in the USA (14.5 culture-confirmed cases per 100,000 population in 2016) [2] have not appreciably declined over the past 15 years [3]. Poultry and poultry meat products are considered some of the main carriers of the organism and represent a significant share of the attributed sources of salmonellosis in humans. Approximately 23.2% of all US outbreak-related salmonellosis cases were attributed to poultry meat with 5.9% specifically linked to turkey in 2020 [4].

The characterization of the occurrence of *Salmonella* along the poultry production chain has been an area of research and policy focus for many years. Joint efforts between policymakers, poultry producers, and industry have reduced the overall *Salmonella* prevalence in poultry products. These efforts have reduced the prevalence of *Salmonella* in ground turkey from 36.6% in 1998 to 15.6% in 2022 [5]. However, prevalence reduction in poultry has not been reflected in a reduced *Salmonella* illness attribution for poultry (19% in 1998–2008 and 23.2% in 2020) [4, 6]. The US Food Safety Inspection Service (FSIS) modified the national *Salmonella* performance standards for ground turkey producers based on contamination rates no greater than 13.5% over a 52-week moving window test period [7]. Despite these efforts, nine *Salmonella* outbreaks attributed to ground turkey products have been reported in the USA since 2010 corresponding to 20.9% of the total number of turkey-related outbreaks reported by the US Centers for Disease Control (CDC) with five being multistate outbreaks that have led to 398 reported illnesses (23.6% of the total reported turkey-related illnesses) [8, 9].

While most of the regulatory efforts have been focused on reducing the overall prevalence of *Salmonella* (presence or absence), very little has been done to estimate the impact on public health by reducing the actual concentration of *Salmonella* in positive lots or by controlling certain high-virulent serotypes. As suggested by other authors, there is already evidence from microbiological risk assessment studies that levels of contamination can be even more important to public health than prevalence as they are directly related to the likelihood that the ingested dose exceeds the infectious dose needed for disease development [10–12]. There is a need to test new performance standards that are based on prevalence and enumeration levels rather than just on absence or presence alone.

Dose–response models have been developed to estimate the relationship between the probability of illness and the ingested *Salmonella* dose in a food product. Models, based on the β-Poisson distribution, have been developed through the use of volunteer and mouse feeding trials (a repository of dose–response models has been created by the Center for Advancing Microbial Risk Assessment (CAMRA), http://camra.msu.edu/) and outbreak data [13]. As pointed out by the authors, dose–response models from feeding trials seem to underestimate the risk of illness by using laboratory-adapted or low-virulent strains not involved in outbreaks. In contrast, dose–response models from outbreak data may overestimate the risk of illness by only accounting for high-virulent serotypes capable of producing outbreaks and ignoring the fact that a high proportion of *Salmonella* serotypes found in food are rarely involved in outbreaks, indicating a low-virulent profile. A combined approach using two different dose–response relationships for high- and low-virulent serotypes could better estimate the true incidence of *Salmonella* human cases due to the consumption of contaminated ground turkey.

The main objective of this study was to develop a risk management framework for *Salmonella* in ground turkey based on the evaluation of public health risk associated with varying concentrations of *Salmonella* contamination and removing lots with high concentration levels and presence of high-virulent serotypes. The probability of outbreak detection at varying *Salmonella* levels and the probability of detection of a positive lot under different testing schemes were also assessed in this study.

## Methodology

### Input data

Prevalence (positive lots) and concentration levels (MPN/g) of *Salmonella* in ground turkey positive lots (2010–2021) were obtained from FSIS samples collected from ground turkey lots processed at federally inspected facilities and retail locations throughout the USA through a Freedom of Information Act (FOIA) request (2022-FSIS-00150-F) (*n* = 8,222 ground turkey samples) (Table 1 and Supplementary Material). Samples below the limit of detection (LOD) (not detected in 325 g) were deemed to be negative and not included in the analysis. *Salmonella*-positive samples with enumeration levels (198 samples) were reported below and above the limit of quantification (LOQ) (0.03 MPN/g). Samples below the LOQ (53.5% of enumerated samples) were characterized by a uniform distribution (Table 1). To characterize the complete distribution of *Salmonella* levels, the mean log of the MPN simulated in each lot (either expressed as a single value from FSIS database or simulated as a uniform distribution when <LOQ) was assigned a within-lot variability (0.38 log MPN/g) [14] and was fitted to a normal distribution (under the assumption of log normality). Overall *Salmonella* concentration level (log MPN/g) simulated by the model (mean, 95% confidence interval (CI)) was assumed to be the overall concentration of all positive ground turkey lots. *Salmonella* serotypes found in positive ground turkey lots were classified as low virulence and high virulence depending on their involvement in human outbreaks as reported by the CDC Annual Report (2006–2016, top 10), and the National Outbreak Reporting System (NORS) reported outbreaks associated with turkey (2000–2020) [2, 9] (Table 1).

**Table 1.** Model inputs

Input variable	Value/Distribution	Source
Model inputs on population and *Salmonella* dose–response data		
National *Salmonella* prevalence ground turkey	15.8% (LOD, not detected in 325 g)	FSIS (2010–2021) (FOIA request)
Characterization-enumerated samples	53.5% < LOQ (0.03 MPN/g)	
	7.5% > 1 MPN/g	
	2.5% > 10 MPN/g	
	0.5% > 100 MPN/g	
Concentration level (log MPN/g)	Samples < LOQ ~ *Uniform* (LOD, LOQ) MPN/g	[14]
	Within-lot variability: 0.38 MPN/g	
	Between-lot variability*: ~Normal (μ_all samples_, σ_between lots_)* truncated at 4 log MPN/g	
Proportion of *Salmonella* high- and low-virulent serotypes^a^	25% (high), 75% (low)	FSIS (2010–2021) (FOIA request)
Proportion of *Salmonella* cells in ground turkey centre point	*~Pert* (0.1,0.16,0.2)	[13]
Serving size (turkey hamburger patty) (g)	*~Pert* (85,113,170)	Industry personal communication
Annual ground turkey production at retail (g)	2.0 × 10^11^	
Total number of ground turkey servings	1.7 × 10^9^	Estimated in this study
*Salmonella* D values (time to reduce 1-log) in ground turkey (min)	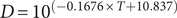	[49]
Dose–response model outbreak data in chicken meat and egg products	Beta-Poisson model	[13]
	*P* (response) 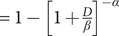	
	where *D* is the ingested dose (CFU), *β* = 51.45, and *α* = 1.3 × 10^−1^	
Dose–response model volunteer feeding trials (*S.* Anatum)	Beta-Poisson model: *β* = 4,730 and *α* = 3.18 × 10^−1^	Center for Advancing Microbial Risk Assessment [28]
Number of cases	Number of contaminated servings × ∫P_illness_	
Underreporting rate	1 out of 29 cases	[1]
Model inputs on consumption patterns at home		
*Consumption of fresh products*		
Proportion of ground turkey consumed at home	94%	Industry personal communication
Proportion of fresh turkey	90%	
Number of servings consumed fresh at home	1.5 × 10^9^	Estimated in this study
Temperature achieved in centre point (fresh turkey burgers cooked at home) (°C)	Histogram ({55.3°C, 93.3°C}, {1,11,10,15,18,24,24,20,17,13,13,8,9,11}) Medium was set as the minimum level of doneness (53.3°C)	[15]
Equivalent cooking time at T_ref_ (min)^b^	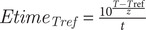 , where *T*_ref_ = 60°C, *D*_60°C_ = 6.73 min, and *z* value = 5.96°C for *Salmonella* in ground turkey	[13, 49]
Reduction after cooking^b^	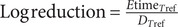	[13]
*Consumption of frozen products*		
Proportion of frozen ground turkey	10%	Industry personal communication
Thawing scenarios	62% (fridge or microwave), 16% (counter), 22% (unthawed)	[15]
Number of servings consumed thawed at home	6.4 × 10^7^	Estimated in this study
Number of servings consumed frozen at home	1.3 × 10^8^	
*Salmonella* reduction after freezing (log CFU/g)	*~Uniform* (0, 0.7)	[29]
Cooking time (fresh turkey hamburger patties) (min)	Cooking time = 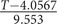 , where *T* is the temperature achieved at the centre point and calculated following the same equation as [15]	
Cooking time (frozen turkey hamburger patties) (min)	*~Pert* (1.0, 1.5, 2.0) × fresh cooking time	[18]
Model inputs on consumption patterns at restaurant		
Proportion of ground turkey servings consumed at restaurants	6%	Industry personal communication
Number of servings consumed at restaurant	3.6 × 10^7^	Estimated in this study
Proportion of restaurants without thermometer (undercooking)	*~Uniform* (0.49, 0.54)	[16, 17]
Proportion of servings and temperature achieved in centre point of turkey hamburger (°C)	*~Uniform* (45.0, 68.2) (under 68.3°C) (5%) *~Pert* (68.4, 80.6, 98.9) (95%)	[16]
Model inputs on cross-contamination		
*Cross-contamination from handling ground and pre-formed patties*		
Proportion consumed as pre-formed patties or ground turkey	74.7%	Estimated in this study [50]
*Cross-contamination from hands to RTE product*		
Proportion of consumers not washing hands	*~Uniform* (0.14, 0.55)	[19–21, 26, 27]
Proportion of consumers preparing a RTE product (i.e. salad)	30%	[20–21]
Microbial amount in raw product available to be transferred	2.2%	[12]
Microbial transfer from raw product to hands	4.0%	[21, 22]
Microbial transfer from hands to RTE product	6.0%	
Effective transfer from hands to RTE product	100%	
*Cross-contamination from hands to mouth*		
Proportion of consumers not washing hands	*~Uniform* (0.14, 0.55)	[19–21, 26, 27]
Proportion of consumers touching bare body parts (i.e. mouth)	1.8%	[19]
Likelihood of hands contact with raw product (log proportion)	*~Normal* (−1.69, 0.81)	[12, 23]
Microbial amount in raw product available to be transferred	2.2%	[12]
Microbial amount in fingertips	6.0%	
Likelihood of transfer from hands to mouth	*~Uniform* (0.34, 0.41)	[12, 24, 25]
*Cross-contamination from board to RTE/cooked product*		
Proportion of consumers not washing the cutting board	*~Uniform* (0, 0.27)	[19–21]
Proportion of consumers preparing a RTE product (i.e. salad)	30%	
Likelihood of board contact with raw product (log proportion)	*~Normal* (−1.45, 1.39)	[12, 23]
Microbial amount in raw product available to be transferred	2.2%	[12]
Likelihood of board contact with RTE product (log proportion)	*~Normal* (−1.42, 0.52)	[12, 23]
Effective transfer from board to cooked/RTE product	100%	[12]
*Cross-contamination from handling self-formed patties*		
Proportion consumed as self-formed patties	25.3%	Estimated in this study
*Cross-contamination from hands to RTE product*		
Proportion of consumers not washing hands	*~Uniform* (0.14, 0.55)	[19–21, 26, 27]
Proportion of consumers preparing a RTE product (i.e. salad)	30%	[20, 21]
Microbial transfer from raw product to hands	4.0%	[21, 22]
Microbial transfer from hands to RTE product	6.0%	
Effective transfer from hands to RTE product	100%	
*Cross-contamination from hands to mouth*		
Proportion of consumers not washing hands	*~Uniform* (0.14, 0.55)	[19–21, 26, 27]
Proportion of consumers touching bare body parts (i.e. mouth)	1.8%	[19]
Likelihood of hands contact with raw product (log proportion)	*~Normal* (−1.69, 0.81)	[12, 23]
Microbial amount in fingertips	6.0%	[12]
Likelihood of transfer from hands to mouth	*~Uniform* (0.34, 0.41)	[12, 24, 25]
*Cross-contamination from cutting board to cooked/RTE product*		
Proportion of consumers not washing the cutting board	*~Uniform* (0, 0.27)	[19–21]
Likelihood of cooked/RTE food contact with contaminated cutting board	30%	
Microbial transfer from cutting board to cooked/RTE product	26%	[21, 22]
Effective transfer from board to cooked/RTE product	46%	

a High-virulent *Salmonella* serotypes implicated in human outbreaks (CDC top 10 from 2006 to 2016) and having turkey as the food vehicle: Braenderup, Enteritidis, Heidelberg, I 4,5,12:i:-, Infantis, Javiana, Muenchen, Newport, and Typhimurium (CDC, 2016; NORS CDC database).

b Same equations were used to estimate the log reduction (log CFU/g) after cooking for the rest of the scenarios.

Data available from scientific literature and provided by industry through personal communication were gathered to characterize the model inputs (Table 1). Serving size was assumed to correspond to a turkey hamburger patty to estimate the total number of ground turkey servings consumed in the US population. Two main consumption scenarios were evaluated separately in the risk assessment model (home and restaurant settings). For the home setting consumption scenario, consumer cooking temperatures surveyed by [15] in beef hamburger patties were used with slight modifications. Modifications were based on assuming consumers were more cautious about the cooking level in turkey, and a minimum of ‘medium level’ of doneness was achieved (reaching a minimum internal temperature of 53.3°C) [16]. For the restaurant consumption scenario, the level of doneness surveyed by [16] in beef hamburger patties was used with some modifications. It was assumed no consumer doneness preferences were requested in the case of turkey hamburger patties, and thus, cooking levels were set by the restaurant. According to [16, 17], only 46–51% of restaurants used a thermometer as a means of checking final food temperatures. In these instances, where final temperatures could be below the FSIS required temperature (71.1°C), cooking profiles were used using [16] where ‘preference was not considered’ (77.8, 45–98.9°C, 95% CI) where 5% of the patties were found to be undercooked (45.0–68.2°C).

Fresh and frozen states were considered to estimate the impact of thawing methods (microwave, room temperature, and refrigerator) on the final *Salmonella* concentration in the home consumption scenario. When it was assumed that consumers cooked products directly from a frozen state, variable cooking times were increased by 50–100% to achieve the same internal temperature as when products were cooked from fresh [18].

**Figure 1.**
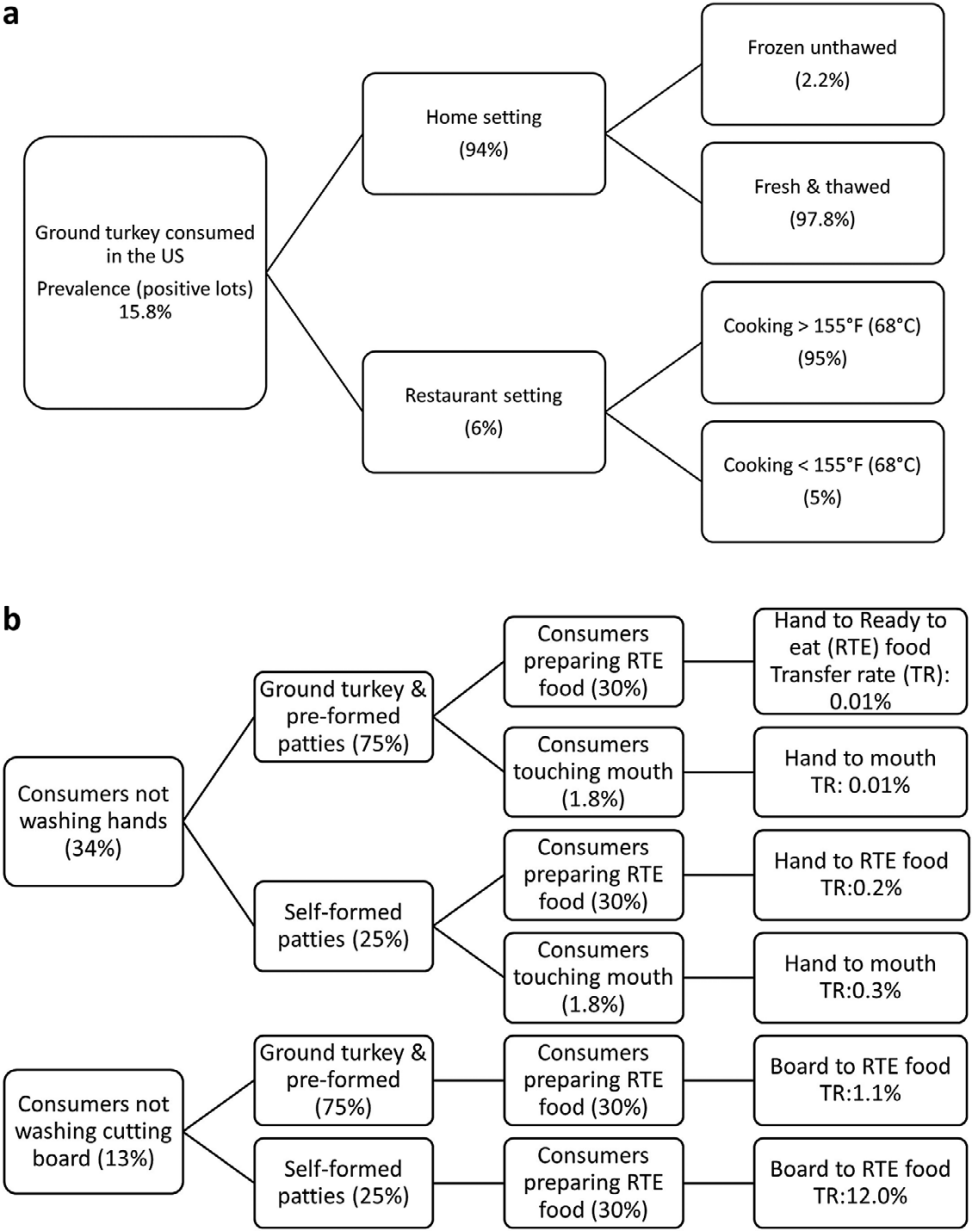
Quantitative risk assessment model framework for *Salmonella* in ground turkey. (a) Model framework. (b) Cross-contamination scenario.

Cross-contamination was modelled separately from the baseline model to account for the number of illnesses occurring due to product mishandling by consumers (Figure 1). Two different cross-contamination scenarios were modelled, the handling of either ground turkey or pre-formed turkey patties (74.7%, assuming less contact with the product) and the handling of self-formed patties (25.3%, assuming higher contact with the product). Each cross-contamination scenario included three different potential microbial transfers from raw ground turkey or patty to (i) an ‘unwashed hand’ to a ready-to-eat (RTE) product (i.e. salad); (ii) an ‘unwashed hand’ to the mouth; and (iii) a ‘contaminated cutting board’ to the cooked product prior to consumption. Each cross-contamination scenario was modelled independently. Microbial transfer coefficients, probability for an effective transfer, and likelihood for the event to occur (i.e. consumers not washing hands) were obtained from consumer observational and pathogen inoculation studies in ground beef, ground pork, and chicken [12, 19–25] (Table 1). The proportion of consumers washing hands or cutting board varied significantly among studies. Ref. [21] reported an average rate of 14 and 27% of consumers not washing hands or cutting board collected from previous studies [20, 26, 27], whereas [19] reported a 55% rate of not washing hands and full compliance with cutting board washing when handling chicken. No cross-contamination was assumed to occur at the frozen stage during the handling of self-formed patties or from the cutting board during the handling of ground and pre-formed patties.

### Dose–response relationship

Dose–response models have been published using volunteer feeding trials and outbreak data to characterize the relationship between the dose ingested and the probability of illness. The first approach has been to use data from feeding trials to characterize the dose–response relationship. The QMRA Wiki (https://qmrawiki.org/) has created a repository of dose–response models by fitting the exponential and β-Poisson models to feeding trial data to find the optimal model. They have estimated dose–response parameters for several *Salmonella* serotypes, namely *Salmonella* Anatum, *Salmonella* Meleagridis, *Salmonella* Newport, and generic non-typhoidal *Salmonella* based on *Salmonella typhimurium.* The second approach has been to use outbreak data that relate the dose ingested with the attack rate. A World Health Organization/Food and Agriculture Organization (WHO/FAO) risk assessment used a β-Poisson model to estimate the number of human cases using *Salmonella* outbreak data in chicken meat and egg products worldwide (Table 1) (FAO/WHO, 2002).

In this study, a combined approach was selected to separate dose–response models for high- and low-virulent serotypes to avoid over- and underestimation. High- and low-virulent criteria were defined to assign dose–response relationships proportionately in the risk scenarios. To estimate the number of salmonellosis cases, high-virulent strains were characterized by the WHO/FAO β-Poisson single-hit model using outbreak data worldwide [13]. Low-virulent strains were characterized by *Salmonella* Anatum dose–response relationship due to being previously found in ground turkey samples tested by FSIS (2022-FSIS-00150-F) and related to isolated human cases [2]. *S.* Anatum dose–response model was characterized by a β-Poisson model estimated by the QMRA Wiki repository using data from volunteer feeding trials [28].

### Risk assessment model framework

A risk assessment model was developed to estimate key public health metrics (predicted number of annual cases and reported cases) in the US population after consumption of contaminated undercooked ground turkey at home and restaurant settings (Figure 1 and Table 1). Data on cooking practices for ground turkey were not available; thus, data on ground beef were used instead assuming a similar thermal profile [29]. Cooking practices observed at the restaurant level were safer (reaching or surpassing Food & Drug Administration (FDA)- and US Department of Agriculture (USDA)-recommended cooking temperatures more frequently) than those reported at home [15, 16]. The stochastic risk assessment model was developed using Excel and @Risk 8.3 (Palisade Corp., Ithaca, NY). Model outputs were obtained by Monte Carlo simulation techniques for 100,000 iterations. During each iteration, a Latin hypercube sampling technique was used to select one random value of each variable or parameter from its respective distribution. Output simulation curves showed a positive skewness and high kurtosis values indicating a long tail to the right (higher values) due to the inherent variability and uncertainty of some of the input variables. Output results were expressed as the mean and 95% CI.

### Public health impact of different risk management strategies

One of the risk management strategies that has been proposed that could lead to a decrease in the number of listeriosis and salmonellosis cases is to include a quantitative microbiological criterion (i.e. <10 CFU/g) and a sampling scheme to test every lot and remove the highly contaminated lots from the market [12, 30–34].

The baseline model estimates were compared with different risk management strategies by assuming the enumeration of *Salmonella* on every positive lot of ground turkey and removing highly contaminated lots (>1 MPN/g and > 10 MPN/g) or following FSIS guidelines of absence/presence of *Salmonella* in 25 g and removing lots with ≥1 MPN per 25 g from the production chain. To estimate the effect of removing positive lots with the presence of high-virulent *Salmonella* serotypes, two approaches were considered. The first approach considered high-virulent serotypes that met both criteria: (i) classified as top 10 due to their involvement in foodborne outbreaks according to the CDC *Salmonella* Annual Report from 2006 to 2016 [2] and (ii) involved in turkey-related outbreaks according to the NORS [9] between 2010 and 2020 (Table 1). The second approach included the serotypes identified by FSIS as highly virulent in poultry products (Enteritidis, Typhimurium, and Infantis) [35]. The baseline model and the different scenarios were run and compared by the impact on the public health metrics (number of illnesses, reported cases) (Tables 2–5).

**Table 2.** Baseline model outputs by using the FAO/WHO dose–response model (assuming no cross-contamination)

Output variable	Mean and 95% CI
Number of illnesses at home (total) (%)	14,080 (11,381–18,663) (87.8%)
Number of illnesses in restaurant (%)	1,956 (1,499–2,485) (12.2%)
Total number of illnesses with low virulence (%)	1,735 (1,442–2,346) (10.8%)
Total number of illnesses with high virulence (%)	14,302 (11,437–18,802) (89.2%)

**Table 3.** Cross-contamination model outputs

Cross-contamination scenario	Transfer scenario	Mean and 95% CI^a^	Proportion of additional illnesses (%)
Ground turkey and pre-formed patties handling	Board	1,846 (85–3,776)	(9.1)
	Hands to mouth	8 (3–12)	0.6
	Hands to RTE product	127 (54–220)	0.04
Self-formed patties handling	Board	623 (32–1,347)	3.1
	Hands to mouth	78 (33–134)	7.9
	Hands to RTE product	1,596 (672–2,792)	0.4

a Additional illnesses to the baseline model corresponding to cross-contamination.

**Table 4.** Public health impact by removing lots with certain concentration levels

Output variable	> 10 MPN/g^a^	> 1 MPN/g^a^	≥ 1 MPN/25 g^a^
Total number of illnesses	9,910 (6,786–11,504)	4,320 (3,666–5,504)	805 (636–1,031)
Total number of reported illnesses	338 (232–393)	147 (125–188)	27 (22–35)
Percentage of reduction with the baseline (number of illnesses)	38.2% (28.3–57.7)	73.1% (65.7–77.1)	95% (93.6–96.0)
Percentage of production lots diverted	0.07% (0.03–0.1)	0.2% (0.2–0.3)	0.8% (0.8–0.9)

a Mean values and 95% confidence intervals.

**Table 5.** Public health impact by removing lots with high-virulent strains

Output variable	CDC top 10 and turkey-related outbreak serotypes^a^	FSIS serotypes^b^
Prevalence	11.8%	13.8%
Percentage of high-virulent serotypes	25.0%	12.7%
Total number of illnesses	2,087 (1,593–2,785)	8,949 (6,698–10,551)
Total number of reported illnesses	71 (54–95)	305 (229–360)
Percentage of reduction with the baseline (number of illnesses)	87.0% (82.6–90.1)	44.2% (34.2–58.2)
Percentage of production lots diverted	3.9%	2.0%

a Braenderup, Enteritidis, Heidelberg, I 4,5,12:i:-, Infantis, Javiana, Muenchen, Newport, and Typhimurium.

b Enteritidis, Typhimurium, and Infantis.

### Lot-by-lot Salmonella testing scheme

A testing scheme was developed to allow the final product microbiological criteria be changed from a moving window of 52 weeks (absence/presence in 325 g, testing weekly samples during 52 weeks of production) to a quantitative three-class mixed plan where positive samples are allowed within a lower and upper bound level [36]. A three-class mixed plan [m defined by the lower microbial limit (absence in 25 or 325 g), M defined by the upper microbial limit (0.1, 1.0, or 2.0 log CFU/g), and c defined as the allowable samples between m and M] was used as the testing scheme to detect lots with a concentration higher than the microbiological criteria proposed in this study (1 and 10 MPN/g) by using the *Salmonella* concentration estimated from FSIS data and ground turkey within-lot variability estimated by industry using *Enterobacteriaceae* as a surrogate for *Salmonella* concentration. Two sample sizes were evaluated by using 25 and 325 g to represent current industry practices (Table 6).

**Table 6.** Performance of a three-class mixed plan for different concentration threshold values (1, 10, and 100 MPN/g) using *Salmonella* spp. concentration in ground turkey

Lot concentration (log MPN/g)	m	M (log MPN/g)	% of positive undetected lots	% of lots above threshold level	*n*	
					*P*_reject_ = 95%, *c* = 1^a^	*P*_reject_ = 95%, *c* = 2^a^
−1.3 ± 1.0 (FSIS samples)^b^	Absence in 25 g: −1.4 log CFU/g	0.1	3.1–3.2	8.1	6	8
		1.0	3.4–4.0	1.2		
		2.0	3.5–4.1	0.06		
	Absence in 325 g: −2.5 log CFU/g	0.1	2.4–4.1	8.1	3	4
		1.0	2.6–4.9	1.2		
		2.0	2.7–5.0	0.06		
−1.3 ± 0.3 (Industry samples)^c^	Absence in 25 g: −1.4 log CFU/g	0.1	0.3–0.5	0.0001	2	3
		1.0		0		
		2.0		0		
	Absence in 325 g: −2.5 log CFU/g	0.1		0.0001		
		1.0		0		
		2.0		0		

a Number of samples to be analysed to detect a positive lot with 95% confidence with one or two samples between *m* and *M.*

b Mean and standard deviation of all FSIS-enumerated samples after 100,000 iterations.

c Within-lot variability based on EB counts of 30 bins (2,000 pounds) of ground turkey (industry personal communication).

### Effect of level of contamination on outbreak detection

**Figure 2.**
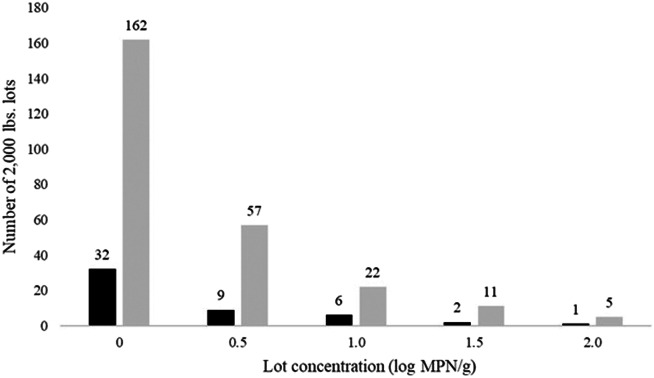
Number of 2,000 Lb. lots for a case or an outbreak of *Salmonella* in ground turkey to be reported by the official surveillance system, by log MPN/g contamination level. Shaded bars correspond to the number of lots for a detectable outbreak. Black bars correspond to the number of lots for a detectable case.

*Salmonella* is a nationally reportable disease, and isolates are routinely submitted to Public Health Laboratories for molecular characterization by whole-genome sequencing (WGS). Individual cases are routinely interviewed to identify risk factors, and when multiple cases are linked by WGS, the case cluster is investigated as a possible outbreak. The likelihood of identifying a common source increases with the number of cases in the cluster and the cluster investigation methods. In a study of *S. enteritidis* clusters defined by WGS, an outbreak source was identified for 13% of clusters of two, 20% of clusters of three, 30% of clusters of four, and 89% of clusters of five or more [37] cases. The number of ground turkey contaminated lots (2,000 lbs.) needed for a single case to be reported by the official surveillance system or for an outbreak source to be identified (five reported cases) was estimated assuming different contamination levels (0–2.0 log MPN/g) (Figure 2).

## Results and discussion

### Overall prevalence, microbial load, and serotype distribution in ground turkey

The overall prevalence of *Salmonella* in ground turkey was 15.8% during the 2010–2021 period (Table 1). The mean *Salmonella* concentration was simulated by the model as −1.3 (−3.3 to 0.7, 95% CI) log MPN/g corresponding to an average concentration of 0.05 MPN/g. Fifty-three per cent of the *Salmonella-*positive ground turkey samples were below the LOQ (0.03 MPN/g), whereas 9.6, 3.1, and 0.5% of the samples were simulated to be higher than 1, 10, and 100 MPN/g, respectively. Several references have also found very low levels of *Salmonella* in poultry products. Ref. [38] found a prevalence level of 14.5% and mean levels of 0.5 log CFU/g in a US commercial turkey production company. Ref. [39] found very low levels of *Salmonella* in chicken carcasses with a mean concentration of 0.02 log CFU/sample (400 mL) at the pre-chilling step, nondetectable levels at the post-chilling step, and 0.07 log CFU/sample in chicken parts in a US broiler commercial processing facility.

The mean ingested dose in the at-home (fresh, thawed, and unthawed) and restaurant (undercooked <68.3°C) consumption scenarios was 0.2, 0.09, 0.8, and 26 MPN, respectively. The mean dose from both consumption scenarios was lower than that reported from outbreak data as [13] estimated an ID_50_ of 40–55 CFU (dose to actively infect 50% of the exposed population) from all *Salmonella* outbreaks combined, and the ingested dose estimated by [40] from *Salmonella typhimurium* 4,5,12:i:- in beef burgers was 315 MPN (142–685, 95% CI).

Serotype distribution in the FSIS 2010–2021 database included Reading (21.0%), Hadar (11.1%), Schwarzengrund (7.3%), Infantis (6.9%), Agona (5.5%), Typhimurium (5.3%), Uganda (5.2%), Senftenberg (4.7%), Muenchen (4.6%), and I 4,5,12:i:- (3.9%). Twenty-five per cent of the positive samples contained serotypes that met the criteria to be considered high-virulent (CDC top 10 and turkey-related) and included Braenderup, Enteritidis, Heidelberg, I 4,5,12:i:-, Infantis, Javiana, Muenchen, Newport, and Typhimurium. Thirteen per cent of the positive samples included the serotypes proposed by FSIS as highly virulent (Enteritidis, Typhimurium, and Infantis).

### Predicted illnesses and reported cases

The baseline model estimated a mean annual number of *Salmonella* cases of 16,036 (12,879–21,148), and the estimated mean number of cases reported to public health officials was 547 (440–722, 95% CI). The majority of illnesses were attributed to high-virulent serotypes (89.2%), whereas most of the consumers were predicted to be sick from consumption at home (87.8%). This is related to different cooking behaviours observed between home and restaurant settings, leading to a greater likelihood of undercooking [15, 16] and the higher number of servings consumed at home.

Cross-contamination scenarios were estimated to produce 4,278 (879–8,281, 95% CI) additional illnesses increasing the total annual illnesses to 20,314 (13,758–29,429, 95% CI) with 693 (469–1,004, 95% CI) reported cases. Illnesses attributed to overall cross-contamination corresponded to 21.1% of the total illnesses where the ‘ground and pre-formed patties’ scenario was attributed to 9.8% whereas the ‘self-formed patties’ scenario was attributed to 11.3% of the total number of illnesses. The cross-contamination scenario with the highest illness attribution was ‘contaminated board’ during the handling of ground and pre-formed patties with 9.1% followed by ‘unwashed hands’ to RTE product during the handling of self-formed patties (Table 3). There is high uncertainty related to the consumer behaviour (i.e. washing hands) when handling poultry products. As noted by [19], there is a high degree of discrepancy between what consumers report to do and what they practice when observed and differences between consumer practices among different observational studies. Finally, all consumer handling studies were performed before the coronavirus disease 2019 (COVID-19) pandemic, which is believed to have significantly increased consumer hygiene practices (i.e. hand washing) [41, 42] with a potential effect on the cross-contamination model assumptions and reduction in the illness estimates.

CDC reports around 50,000 *Salmonella* laboratory-confirmed cases (46,623 for 2016) (CDC, 2018). Using [1] underreporting factor (29.3) and percentage of foodborne (94%) will equal a total of 1,284,091 *Salmonella* cases occurring annually in the USA. Assuming that 5.9% of non-typhoidal *Salmonella* cases in the USA are attributable to turkey [4] that corresponds to 75,761 estimated illnesses. Total turkey production according to the latest World Agricultural Supply and Demand Estimates (WASDE) report was 2,671,203 metric tons [43]. Total ground turkey sold in the USA was estimated at 203,318 metric tons, corresponding to 7.2% of total turkey production (industry personal communication). As mentioned earlier, [9] database reports 23.6% of the total reported turkey-related illnesses attributed to ground turkey. Based on this calculation, the ground turkey-related illnesses would be in the range of 17,500 illnesses (604 reported cases) using the Interagency Food Safety Analytics Collaboration (IFSAC) estimates (75,761 total turkey-related illnesses). Our model estimates for ground turkey-related illnesses (20,314 illnesses, 26.8% ground turkey attribution of total turkey-related illnesses) were aligned to the CDC and IFSAC ground turkey-related illness highlighting the fact that ground turkey represents a higher risk than most other turkey products.

### Public health impact of risk management strategies

The effect of removing lots with higher *Salmonella* concentration levels was evaluated by assuming every positive lot was enumerated for *Salmonella,* and if the concentration was found to be higher than a threshold level, it was removed from the food production and thermally processed. FSIS data showed that 3.1% of enumerated positive samples contained a concentration higher than 10 MPN/g. Removing lots with a microbial load >10 MPN/g reduced the overall *Salmonella* prevalence to 15.7% and mean concentration to −1.4 (−3.2 to 0.3, 95% CI) log MPN/g. Through the removal of these lots above the 10 MPN/g threshold, the model predicted 9,910 illnesses that would lead to 338 reported cases, which is a reduction of 38.2% in the mean number of illnesses of the baseline model (Table 4). Removing lots exceeding 1 MPN/g reduced the overall prevalence to 15.6% and mean concentration to −1.5 (−3.1 to −0.05, 95% CI) log MPN/g leading to a reduction of 73.1% of the mean number of illnesses. Applying a more stringent microbial threshold as the absence of *Salmonella* in 25 g, the model predicts that the prevalence of contamination would be reduced to 14.9% and the mean concentration to −1.9 (−2.8 to −1.0, 95% CI) log MPN/g, resulting in a net reduction of 95.0% of illnesses. Although the absence of *Salmonella* in 25 g guideline would reduce the greatest number of illnesses, it would also reduce the availability of fresh ground turkey. An estimated 0.8% of production lots would need to be diverted to a thermal process, or potentially wasted, whereas only between 0.07 and 0.2% would be removed or diverted if 10 or 1 MPN/g guidelines were used (Table 4).

The effect of removing lots above the 10 MPN/g on cross-contamination-related illnesses was estimated as 43.3–62.5%, whereas a reduction of 65.8–87.5% was estimated by removing lots higher than 1 MPN/g. The ‘hand-to-mouth’ transfer when handling ground and pre-formed patties was the route of transmission with the highest illness reduction.

The net effect of reducing the overall mean concentration of a pathogen within a production lot on reduction in number of illnesses has been evaluated in previous risk assessment studies of *Salmonella* in poultry and pork products [12, 20, 44], indicating that concentration of contamination and consumer education on adequate handling and cooking practices have the greatest impact on public health, and efforts at any stage of production or processing that reduces the level of *Salmonella* on the end product will reduce risk to a greater extent.

The effect of removing positive lots with the presence of high-virulent serotypes is shown in Table 5. When the top 10 serotypes according to CDC human outbreak data and turkey-related outbreak-causing serotypes were excluded, the prevalence was reduced to 11.8% and the total number of estimated illnesses was estimated as 2,087 (71 reported cases). This represents a reduction of 87% over the baseline estimates. If the serotypes proposed by FSIS were excluded, the effect on prevalence and total illnesses was lower, reducing the *Salmonella* prevalence to 13.8% of positive lots and percentage of highly virulent serotypes to 12% of the lots. When the highly virulent serotypes proposed by FSIS were removed, the number of illnesses was reduced by 44.2% from baseline. However, the number of lots needed to be diverted to thermal process was lower than removing lots with high-virulent serotypes according to the CDC, reaching 2.0%.

The testing scheme proposed in the present study (three-class mixed plan) was evaluated to detect lots with mean *Salmonella* concentrations higher than 1 and 10 MPN/g (Table 6). The percentage of lots higher than the 1 MPN/g threshold level by using FSIS *Salmonella* enumeration data was 8.1%, whereas 1.2% were higher than 10 MPN/g and 0.06% were higher than 100 MPN/g. The number of samples required to be analysed to detect a contaminated lot with 95% confidence with m (absence in 25 g) and M (0.1, 1.0, or 2.0 log CFU/g) was six samples when *c* = 1 (only one sample allowed to be between m and M) and eight samples when *c* = 2 (Table 6). When the sample size was increased to 325 g to follow FSIS testing requirements (m, absence in 325 g), the number of samples required to be analysed was reduced to 3 when *c* = 1 and 4 when *c* = 2 (Table 6). A second testing scenario used within-lot variability of *Enterobacteriaceae* as a surrogate based on ground turkey industry data. In this scenario, a very small fraction of the lots was higher than the threshold levels. The number of samples needed to detect a lot with 95% confidence was also reduced to two samples when *c* = 1 and 3 samples when *c* = 2. As with any sampling plan, certain positive lots could remain undetected. The percentage of positive undetected lots, or ‘false negative lots’, ranged from 2.4 to 5.0 and 0.3 to 0.5% when using FSIS or industry data, respectively (Table 6). These lots could contain a higher concentration than the threshold level and reach the market.

### Effect of level of contamination and number of lots on outbreak detection

The industry usually uses 2,000 lb. (907 kg) lots of ground turkey in their daily production schemes in the USA (industry personal communication). Figure 2 shows the number of contaminated ground turkey lots needed for one detectable case (reported case) and the number of lots needed for one detectable outbreak (five reported cases) at various contamination levels (1–100 MPN/g) (Figure 2). When the *Salmonella* mean concentration level was estimated as 1 MPN/g (0 log MPN/g), the number of contaminated lots ranged from 32 for one detectable case to 162 for a detectable outbreak (five reported *Salmonella* cases) (Figure 2). At contamination levels of 100 MPN/g, only five contaminated lots would be needed for a detectable outbreak (Figure 2). In the two most recent outbreaks of salmonellosis associated with ground turkey in the USA, seven cases were associated with a recall of approximately 35,380 kg (78,000 lbs.) of ground turkey in 2019, and 33 cases were associated with approximately 95,707 kg (211,000 lbs.) of ground turkey in 2021 [45–48]. Imputed levels of contamination would have been between 10 and 32 MPN/g (1.0–1.5 log MPN/g) in these outbreaks based on the results of our risk assessment model. These data suggest that a high proportion of outbreaks and poultry-associated sporadic infections are attributable to products with relatively high levels of *Salmonella* contamination.

## Conclusions

Different dose–response models for high- and low-virulent serotypes were used to estimate the annual number of salmonellosis illnesses and reported cases from the consumption of contaminated ground turkey. This combined approach allowed balancing under- and over-reporting. Removing highly contaminated lots and highly virulent serotypes could reduce the occurrence of illnesses and the notifiable number of outbreaks. Risk management strategies focused on interventions that can reduce *Salmonella* load to low levels of contamination will have great public health benefits while avoiding costs associated with the destruction of products with detectable but low levels. Introducing lot-by-lot testing under current regulatory schemes and defining allowable quantitative regulatory limits will improve the availability of *Salmonella* enumeration data and compliance with food safety standards by the poultry industry. Ideally, regulatory efforts in food safety should link public health metrics with quantifiable food safety metrics based on the results of a risk assessment.

## Supporting information

Sampedro et al. supplementary material Sampedro et al. supplementary material

## Data Availability

The risk assessment model that supports the findings of this study can be accessed in the supplementary material.

## Supplementary material

For supplementary material accompanying this paper visit http://doi.org/10.1017/S0950268823002029. click here to view supplementary material
